# Supplementary material for: Single-Cell Transcriptomics Reveals the Expression of Aging- and Senescence-Associated Genes in Distinct Cancer Cell Populations
Source: Cells. 2021 Nov 11;10(11):3126. doi: 10.3390/cells10113126 (PMC8623328; doi:10.3390/cells10113126)
Supplement: Supplementary file 1 [file cells-10-03126-s001.zip › cells-1409365-supplementary.pdf]

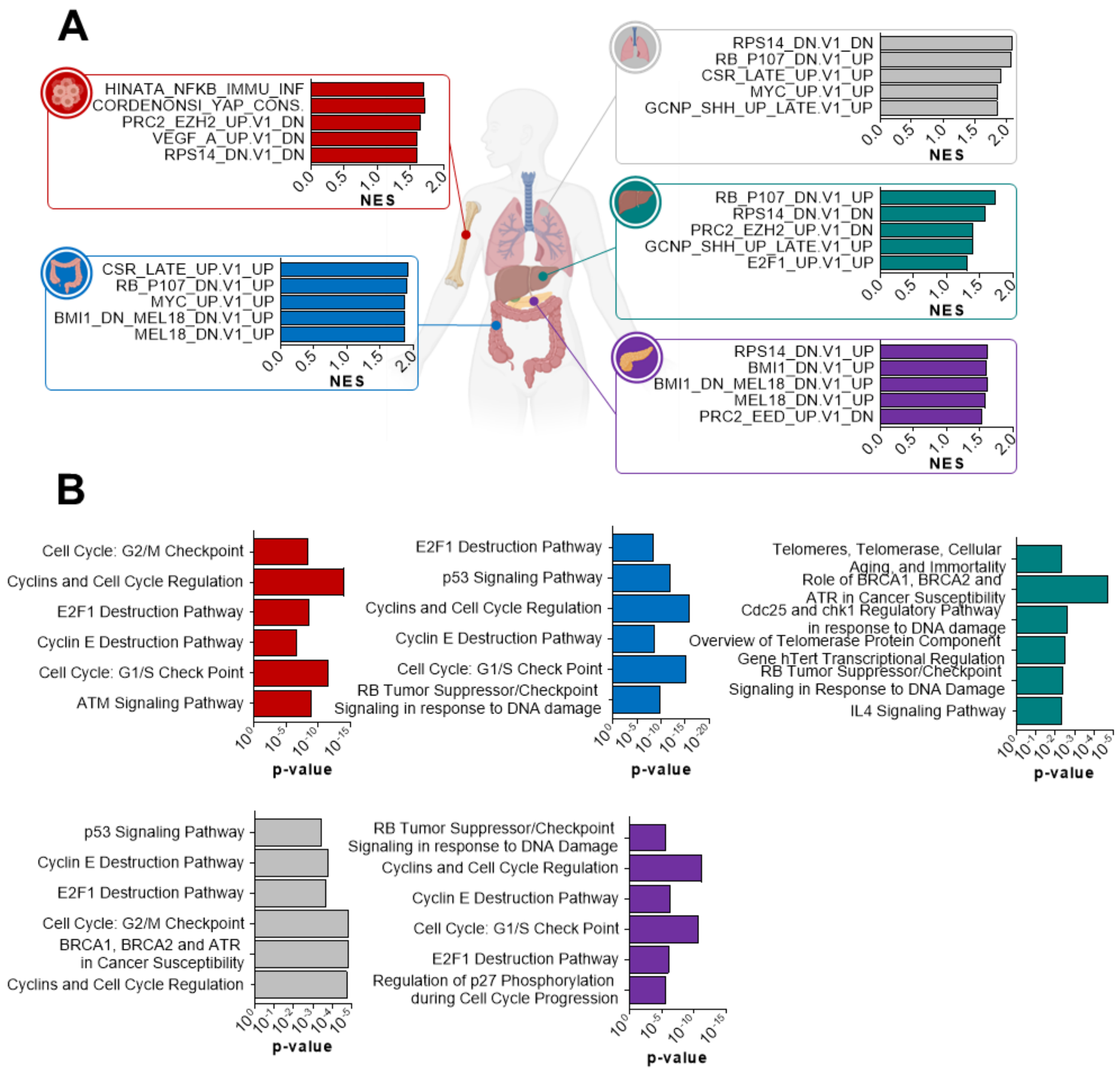

Supplementary Figure S1: Enriched oncogenic signatures per cancer entity. (A) GSEA was performed to identify highly enriched oncogenic signatures (C6) for publicly available CML, CRC, HCC, LC and PDAC bulk mRNA-seq datasets. The top five enriched pathways were displayed per cancer entity [35,37,39,40,42–44]. Raw data is provided in Supplementary Table S2. (B) ASIGs upregulated in cancer were analyzed using gene ontology (Enrichr, BioCarta 2016 [61]). Parts of panel A were created with BioRender.com.

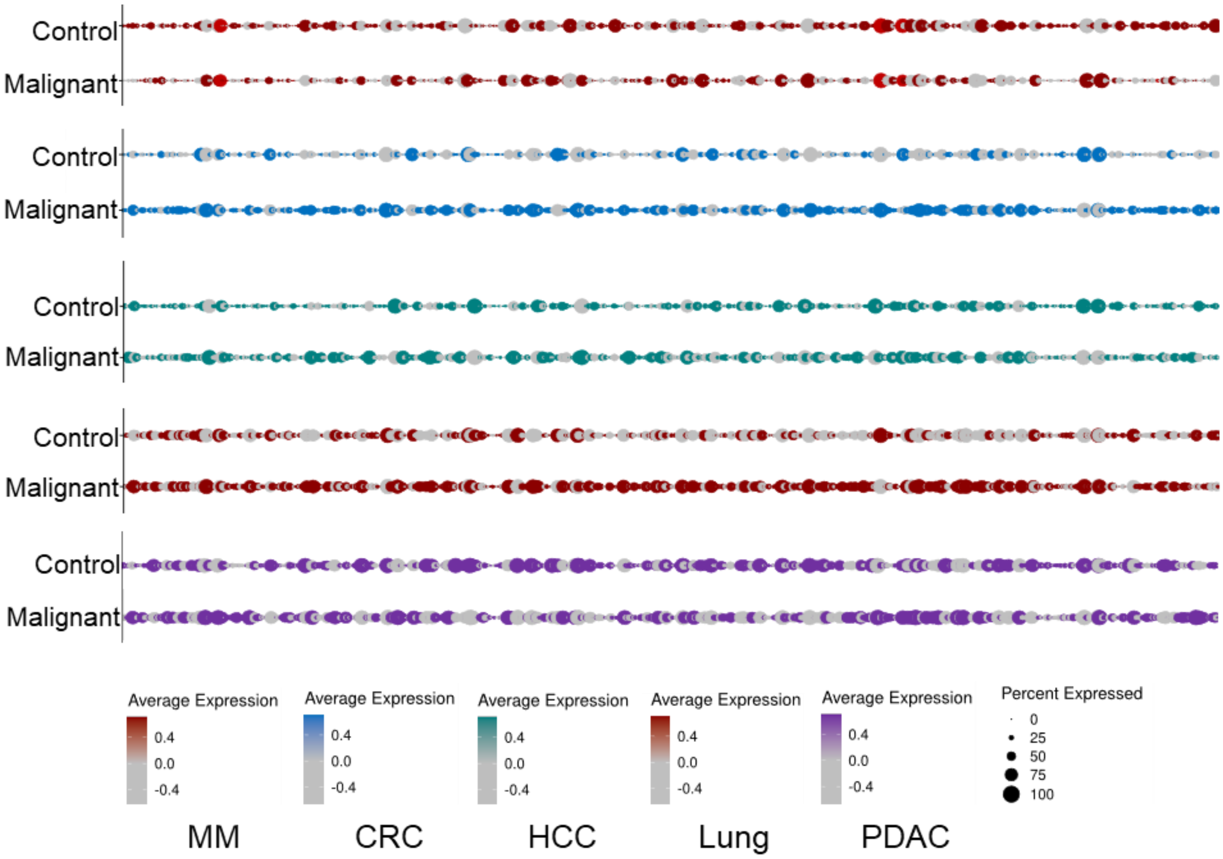

Supplementary Figure S2: Expression of 1,153 ASIGs in malignant and control cells. Each dot represents one ASIG, the expression level is shown via color intensity and the percentage of cells expressing the respective gene is represented by dot size. Epithelial CRC and LC cells displayed a marked increase in ASIG expression in malignant compared to control cells while in MM, HCC and PDAC no major differences were detected.

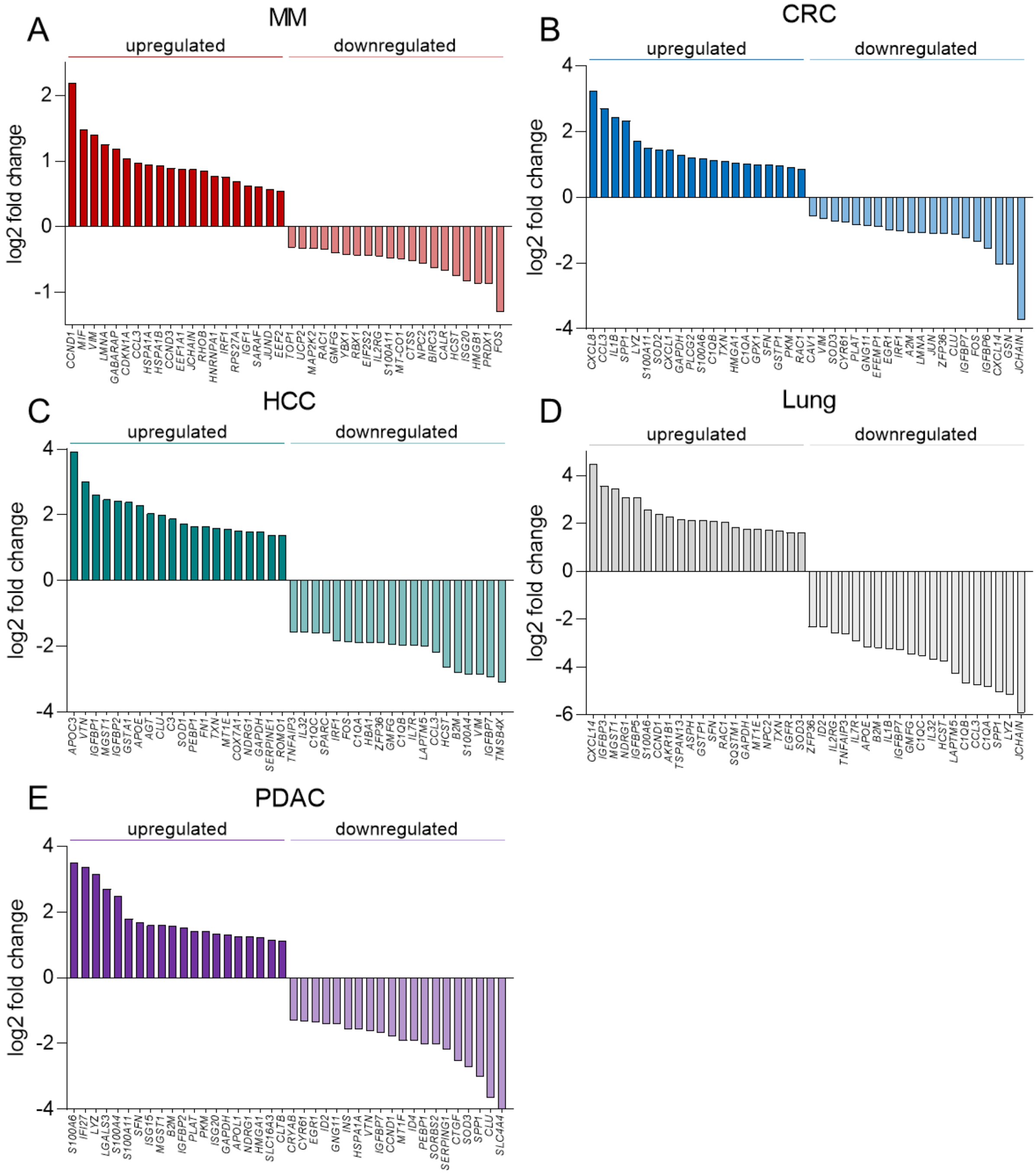

Supplementary Figure S3: Top 20 up- and downregulated ASIGs in malignant cells extracted from scRNA-seq data in each cancer entity. In each scRNA-seq dataset, the top 20 down- and upregulated genes in malignant compared to control cells were presented based on their log_2 fold changes (padj <0.01) in (A) MM, (B) CRC, (C) HCC, (D) LC, (E) PDAC.

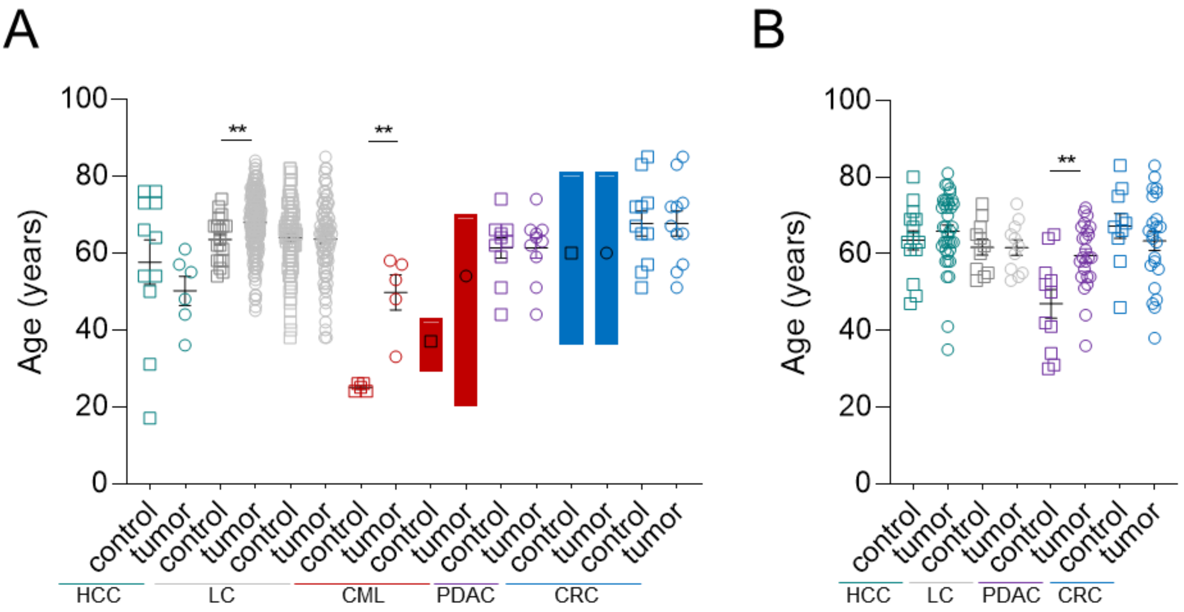

Supplementary Figure S4: Patients' age in bulk RNA-seq and scRNA-seq studies. (A) Out of the publicly available bulk RNA-seq datasets analyzed in this manuscript, two studies did not provide patients' age (paired samples in HCC GSE105130, unpaired samples in PDAC E-MTAB-3494). For the first LC dataset (GSE81089) the difference was 4.4 years between the cohorts: 63.53 years in control vs. 67.93 years in tumor, Mann-Whitney test p=0.0079. In the first CML dataset, there was a 24.8-year difference (GSE100026; control: 25 years vs. tumor: 49.8 years, Mann-Whitney test=0.0079). In the CML (GSE144119) and CRC (GSE50760) datasets, the range instead of the single patient characteristics was provided in the original studies (leading to a depiction as bar graph with median). (B) In the single cell datasets, the samples in control and tumor were of comparable age, except for the PDAC dataset (PDAC PRJCA001063, control: 47.0 years vs. tumor:59.54 years, unpaired t-test p=0.0015). For the CML dataset (GSE175385), the age was not provided. D'Agostino & Pearson test for normality. If passed, an unpaired t-test was performed. Otherwise, a Mann-Whitney test was performed. *p<0.05, **p<0.01, ***p<0.001.
