# Supplementary material for: Single-Cell Transcriptomics Reveals the Expression of Aging- and Senescence-Associated Genes in Distinct Cancer Cell Populations
Source: Cells. 2021 Nov 11;10(11):3126. doi: 10.3390/cells10113126 (PMC8623328; doi:10.3390/cells10113126)
Supplement: Supplementary file 1 [file cells-10-03126-s001.zip › RNA_seq_PDAC.nb.html]

R Notebook RNA Seq complete


Code 

- Show All Code
- Hide All Code
- Download Rmd

# R Notebook RNA Seq complete


```
#load all necessary libraries (some might need installing via BiocManager, follow BiocManager::install("PACKAGE") )
packages = c("BiocManager", "gtools", "caTools") 

package.check <- lapply(
    packages,
    FUN = function(x) {
        
if (!require(x, character.only = TRUE)) {
            install.packages(x, dependencies = TRUE)
            library(x, character.only = TRUE)
        }
    }
)


packages = c("gplots", "DESeq2", "readxl", "dplyr", "utils", "ggplot2", "ggrepel")


#if the packages do not exist, they will be installed:

package.check <- lapply(
    packages,
    FUN = function(x) {
        
if (!require(x, character.only = TRUE)) {
            BiocManager::install(x, dependencies = TRUE)
            library(x, character.only = TRUE)
        }
    }
)


#create a new folder for this dataset, and set the working directory to this folder
dir.create("GSE119794")
setwd("GSE119794")

#get the raw_counts_file from GEO, download them and untar them
options(timeout=100000000000)
url="https://www.ncbi.nlm.nih.gov/geo/download/?acc=GSE119794&format=file"
download.file(url, "GSE119794_RAW.tar")
untar("GSE119794_RAW.tar")

#just read in all files that are needed for your analyses (in this case, just normal and tumor RNA-seq data is needed, not the miRNA data)
all_files <- list.files(path = ".")[2:21]
for (i in 1:length(all_files)) {
    nam <- paste(all_files[i], sep = " ")
    assign(nam, read.delim(all_files[i], stringsAsFactors=FALSE))
}

#get the list of all your files in the new folder
DF_obj <- lapply(ls(), get)

#create a merged table with all these files
jointdataset <- merge(DF_obj[2], DF_obj[3:21], all.y=TRUE)
#delete all duplications of the gene names
df_sub <- select(jointdataset, -contains("X."))
rawcounts <- df_sub
#remove all "helping" files
rm(list = ls()[!ls() %in% c("rawcounts")])
write.csv(rawcounts, "rawcounts.csv")

#You now created a file named rawcounts, in this example from GSE119794. This file should be in your working directory. The  first row contains either "normal" or "tumor", while the first column contains the gene name (or gene symbol). A1 is empty.
# apply your experimental setup. There are 20 samples, 10 of each for each "condition"
design1<-data.frame(experiment=colnames(rawcounts[,2:21]),  treatment = c("normal", "normal", "normal", "normal", "normal", "normal", "normal", "normal", "normal", "normal", "tumor", "tumor", "tumor", "tumor", "tumor", "tumor", "tumor", "tumor", "tumor", "tumor"))
# add the rownames to the design
rownames(design1)<-colnames(rawcounts[,2:21])
# transform the rawcoutns into a Matrix, according to your experimental setup
normalandtumor <- DESeqDataSetFromMatrix(countData = (round(rawcounts[,2:21])), colData = design1, design = ~  treatment)
# perform the actual DESeq
dnormalandtumor <- DESeq(normalandtumor)
# create a DE results table (the standard parameters are lfcThreshold=0, alpha=0.1, minmu=0.5). The p-value correction is performed according to the methods of Benjamini and Hochberg.
normalandtumor_pdac_res <- results(dnormalandtumor,contrast=c("treatment","normal", "tumor"))
# name the results table according to your excel-sheet
normalandtumor_pdac_res@rownames <- rawcounts$X
# write the results as a *.csv file.Note: high log2FC refers to a high expression in the first condition that is named in the results command (here: "normal")
write.csv(normalandtumor_pdac_res, file="normal_vs_tumor_pdac.csv", row.names = rawcounts$X)

results <- as.data.frame(normalandtumor_pdac_res)
results=results[order(results$padj),]

ggplot(results, aes(x=log2FoldChange, y=-log10(padj))) +
    geom_point(aes(color="grey", size=1.5, alpha=0.4)) +
    ggtitle('Volcano Plot') +
    labs(y=expression('-Log'[10]*' P'[adj]), x=expression('Log'[2]*' fold change')) +
    theme_minimal() +
    geom_text_repel(data=results[1:10,], aes(x = log2FoldChange, y = -log10(padj), label=rownames(results)[1:10]))+
    theme(legend.position="none", plot.title = element_text(size = rel(1.5), hjust = 0.5))+
    geom_point(data=results[1:10,],aes(x = log2FoldChange, y = -log10(padj), color="red", size=1.5, alpha=0.4))+
    scale_color_manual(values=c('gray','red'))

ggsave(
    "volcanoplot.png",
    plot = last_plot(),
    dpi=600)

#normalize the rawcounts, and write a table with the normalized counts
dnormalandtumor_VSD <-varianceStabilizingTransformation(dnormalandtumor)
write.csv(assay(dnormalandtumor_VSD), file="normalized_counts.csv", row.names = rawcounts$X)

#make a small PCA plot for an initial overview
dnormalandtumor_VSD
plotPCA(dnormalandtumor_VSD,ntop=30000,intgroup="treatment")+ theme_bw()

ggsave(
    "PCAplot.png",
    plot = last_plot(),
    dpi=600)

print("done")
```


LS0tCnRpdGxlOiAiUiBOb3RlYm9vayBSTkEgU2VxIGNvbXBsZXRlIgpvdXRwdXQ6IGh0bWxfbm90ZWJvb2sKLS0tCgpgYGB7cn0KI2xvYWQgYWxsIG5lY2Vzc2FyeSBsaWJyYXJpZXMgKHNvbWUgbWlnaHQgbmVlZCBpbnN0YWxsaW5nIHZpYSBCaW9jTWFuYWdlciwgZm9sbG93IEJpb2NNYW5hZ2VyOjppbnN0YWxsKCJQQUNLQUdFIikgKQpwYWNrYWdlcyA9IGMoIkJpb2NNYW5hZ2VyIiwgImd0b29scyIsICJjYVRvb2xzIikgCgpwYWNrYWdlLmNoZWNrIDwtIGxhcHBseSgKICAgIHBhY2thZ2VzLAogICAgRlVOID0gZnVuY3Rpb24oeCkgewogICAgICAgIAppZiAoIXJlcXVpcmUoeCwgY2hhcmFjdGVyLm9ubHkgPSBUUlVFKSkgewogICAgICAgICAgICBpbnN0YWxsLnBhY2thZ2VzKHgsIGRlcGVuZGVuY2llcyA9IFRSVUUpCiAgICAgICAgICAgIGxpYnJhcnkoeCwgY2hhcmFjdGVyLm9ubHkgPSBUUlVFKQogICAgICAgIH0KICAgIH0KKQoKCnBhY2thZ2VzID0gYygiZ3Bsb3RzIiwgIkRFU2VxMiIsICJyZWFkeGwiLCAiZHBseXIiLCAidXRpbHMiLCAiZ2dwbG90MiIsICJnZ3JlcGVsIikKCgojaWYgdGhlIHBhY2thZ2VzIGRvIG5vdCBleGlzdCwgdGhleSB3aWxsIGJlIGluc3RhbGxlZDoKCnBhY2thZ2UuY2hlY2sgPC0gbGFwcGx5KAogICAgcGFja2FnZXMsCiAgICBGVU4gPSBmdW5jdGlvbih4KSB7CiAgICAgICAgCmlmICghcmVxdWlyZSh4LCBjaGFyYWN0ZXIub25seSA9IFRSVUUpKSB7CiAgICAgICAgICAgIEJpb2NNYW5hZ2VyOjppbnN0YWxsKHgsIGRlcGVuZGVuY2llcyA9IFRSVUUpCiAgICAgICAgICAgIGxpYnJhcnkoeCwgY2hhcmFjdGVyLm9ubHkgPSBUUlVFKQogICAgICAgIH0KICAgIH0KKQoKCiNjcmVhdGUgYSBuZXcgZm9sZGVyIGZvciB0aGlzIGRhdGFzZXQsIGFuZCBzZXQgdGhlIHdvcmtpbmcgZGlyZWN0b3J5IHRvIHRoaXMgZm9sZGVyCmRpci5jcmVhdGUoIkdTRTExOTc5NCIpCnNldHdkKCJHU0UxMTk3OTQiKQoKI2dldCB0aGUgcmF3X2NvdW50c19maWxlIGZyb20gR0VPLCBkb3dubG9hZCB0aGVtIGFuZCB1bnRhciB0aGVtCm9wdGlvbnModGltZW91dD0xMDAwMDAwMDAwMDApCnVybD0iaHR0cHM6Ly93d3cubmNiaS5ubG0ubmloLmdvdi9nZW8vZG93bmxvYWQvP2FjYz1HU0UxMTk3OTQmZm9ybWF0PWZpbGUiCmRvd25sb2FkLmZpbGUodXJsLCAiR1NFMTE5Nzk0X1JBVy50YXIiKQp1bnRhcigiR1NFMTE5Nzk0X1JBVy50YXIiKQoKI2p1c3QgcmVhZCBpbiBhbGwgZmlsZXMgdGhhdCBhcmUgbmVlZGVkIGZvciB5b3VyIGFuYWx5c2VzIChpbiB0aGlzIGNhc2UsIGp1c3Qgbm9ybWFsIGFuZCB0dW1vciBSTkEtc2VxIGRhdGEgaXMgbmVlZGVkLCBub3QgdGhlIG1pUk5BIGRhdGEpCmFsbF9maWxlcyA8LSBsaXN0LmZpbGVzKHBhdGggPSAiLiIpWzI6MjFdCmZvciAoaSBpbiAxOmxlbmd0aChhbGxfZmlsZXMpKSB7CiAgICBuYW0gPC0gcGFzdGUoYWxsX2ZpbGVzW2ldLCBzZXAgPSAiICIpCiAgICBhc3NpZ24obmFtLCByZWFkLmRlbGltKGFsbF9maWxlc1tpXSwgc3RyaW5nc0FzRmFjdG9ycz1GQUxTRSkpCn0KCiNnZXQgdGhlIGxpc3Qgb2YgYWxsIHlvdXIgZmlsZXMgaW4gdGhlIG5ldyBmb2xkZXIKREZfb2JqIDwtIGxhcHBseShscygpLCBnZXQpCgojY3JlYXRlIGEgbWVyZ2VkIHRhYmxlIHdpdGggYWxsIHRoZXNlIGZpbGVzCmpvaW50ZGF0YXNldCA8LSBtZXJnZShERl9vYmpbMl0sIERGX29ialszOjIxXSwgYWxsLnk9VFJVRSkKI2RlbGV0ZSBhbGwgZHVwbGljYXRpb25zIG9mIHRoZSBnZW5lIG5hbWVzCmRmX3N1YiA8LSBzZWxlY3Qoam9pbnRkYXRhc2V0LCAtY29udGFpbnMoIlguIikpCnJhd2NvdW50cyA8LSBkZl9zdWIKI3JlbW92ZSBhbGwgImhlbHBpbmciIGZpbGVzCnJtKGxpc3QgPSBscygpWyFscygpICVpbiUgYygicmF3Y291bnRzIildKQp3cml0ZS5jc3YocmF3Y291bnRzLCAicmF3Y291bnRzLmNzdiIpCgojWW91IG5vdyBjcmVhdGVkIGEgZmlsZSBuYW1lZCByYXdjb3VudHMsIGluIHRoaXMgZXhhbXBsZSBmcm9tIEdTRTExOTc5NC4gVGhpcyBmaWxlIHNob3VsZCBiZSBpbiB5b3VyIHdvcmtpbmcgZGlyZWN0b3J5LiBUaGUgIGZpcnN0IHJvdyBjb250YWlucyBlaXRoZXIgIm5vcm1hbCIgb3IgInR1bW9yIiwgd2hpbGUgdGhlIGZpcnN0IGNvbHVtbiBjb250YWlucyB0aGUgZ2VuZSBuYW1lIChvciBnZW5lIHN5bWJvbCkuIEExIGlzIGVtcHR5LgojIGFwcGx5IHlvdXIgZXhwZXJpbWVudGFsIHNldHVwLiBUaGVyZSBhcmUgMjAgc2FtcGxlcywgMTAgb2YgZWFjaCBmb3IgZWFjaCAiY29uZGl0aW9uIgpkZXNpZ24xPC1kYXRhLmZyYW1lKGV4cGVyaW1lbnQ9Y29sbmFtZXMocmF3Y291bnRzWywyOjIxXSksICB0cmVhdG1lbnQgPSBjKCJub3JtYWwiLCAibm9ybWFsIiwgIm5vcm1hbCIsICJub3JtYWwiLCAibm9ybWFsIiwgIm5vcm1hbCIsICJub3JtYWwiLCAibm9ybWFsIiwgIm5vcm1hbCIsICJub3JtYWwiLCAidHVtb3IiLCAidHVtb3IiLCAidHVtb3IiLCAidHVtb3IiLCAidHVtb3IiLCAidHVtb3IiLCAidHVtb3IiLCAidHVtb3IiLCAidHVtb3IiLCAidHVtb3IiKSkKIyBhZGQgdGhlIHJvd25hbWVzIHRvIHRoZSBkZXNpZ24Kcm93bmFtZXMoZGVzaWduMSk8LWNvbG5hbWVzKHJhd2NvdW50c1ssMjoyMV0pCiMgdHJhbnNmb3JtIHRoZSByYXdjb3V0bnMgaW50byBhIE1hdHJpeCwgYWNjb3JkaW5nIHRvIHlvdXIgZXhwZXJpbWVudGFsIHNldHVwCm5vcm1hbGFuZHR1bW9yIDwtIERFU2VxRGF0YVNldEZyb21NYXRyaXgoY291bnREYXRhID0gKHJvdW5kKHJhd2NvdW50c1ssMjoyMV0pKSwgY29sRGF0YSA9IGRlc2lnbjEsIGRlc2lnbiA9IH4gIHRyZWF0bWVudCkKIyBwZXJmb3JtIHRoZSBhY3R1YWwgREVTZXEKZG5vcm1hbGFuZHR1bW9yIDwtIERFU2VxKG5vcm1hbGFuZHR1bW9yKQojIGNyZWF0ZSBhIERFIHJlc3VsdHMgdGFibGUgKHRoZSBzdGFuZGFyZCBwYXJhbWV0ZXJzIGFyZSBsZmNUaHJlc2hvbGQ9MCwgYWxwaGE9MC4xLCBtaW5tdT0wLjUpLiBUaGUgcC12YWx1ZSBjb3JyZWN0aW9uIGlzIHBlcmZvcm1lZCBhY2NvcmRpbmcgdG8gdGhlIG1ldGhvZHMgb2YgQmVuamFtaW5pIGFuZCBIb2NoYmVyZy4Kbm9ybWFsYW5kdHVtb3JfcGRhY19yZXMgPC0gcmVzdWx0cyhkbm9ybWFsYW5kdHVtb3IsY29udHJhc3Q9YygidHJlYXRtZW50Iiwibm9ybWFsIiwgInR1bW9yIikpCiMgbmFtZSB0aGUgcmVzdWx0cyB0YWJsZSBhY2NvcmRpbmcgdG8geW91ciBleGNlbC1zaGVldApub3JtYWxhbmR0dW1vcl9wZGFjX3Jlc0Byb3duYW1lcyA8LSByYXdjb3VudHMkWAojIHdyaXRlIHRoZSByZXN1bHRzIGFzIGEgKi5jc3YgZmlsZS5Ob3RlOiBoaWdoIGxvZzJGQyByZWZlcnMgdG8gYSBoaWdoIGV4cHJlc3Npb24gaW4gdGhlIGZpcnN0IGNvbmRpdGlvbiB0aGF0IGlzIG5hbWVkIGluIHRoZSByZXN1bHRzIGNvbW1hbmQgKGhlcmU6ICJub3JtYWwiKQp3cml0ZS5jc3Yobm9ybWFsYW5kdHVtb3JfcGRhY19yZXMsIGZpbGU9Im5vcm1hbF92c190dW1vcl9wZGFjLmNzdiIsIHJvdy5uYW1lcyA9IHJhd2NvdW50cyRYKQoKcmVzdWx0cyA8LSBhcy5kYXRhLmZyYW1lKG5vcm1hbGFuZHR1bW9yX3BkYWNfcmVzKQpyZXN1bHRzPXJlc3VsdHNbb3JkZXIocmVzdWx0cyRwYWRqKSxdCgpnZ3Bsb3QocmVzdWx0cywgYWVzKHg9bG9nMkZvbGRDaGFuZ2UsIHk9LWxvZzEwKHBhZGopKSkgKwogICAgZ2VvbV9wb2ludChhZXMoY29sb3I9ImdyZXkiLCBzaXplPTEuNSwgYWxwaGE9MC40KSkgKwogICAgZ2d0aXRsZSgnVm9sY2FubyBQbG90JykgKwogICAgbGFicyh5PWV4cHJlc3Npb24oJy1Mb2cnWzEwXSonIFAnW2Fkal0pLCB4PWV4cHJlc3Npb24oJ0xvZydbMl0qJyBmb2xkIGNoYW5nZScpKSArCiAgICB0aGVtZV9taW5pbWFsKCkgKwogICAgZ2VvbV90ZXh0X3JlcGVsKGRhdGE9cmVzdWx0c1sxOjEwLF0sIGFlcyh4ID0gbG9nMkZvbGRDaGFuZ2UsIHkgPSAtbG9nMTAocGFkaiksIGxhYmVsPXJvd25hbWVzKHJlc3VsdHMpWzE6MTBdKSkrCiAgICB0aGVtZShsZWdlbmQucG9zaXRpb249Im5vbmUiLCBwbG90LnRpdGxlID0gZWxlbWVudF90ZXh0KHNpemUgPSByZWwoMS41KSwgaGp1c3QgPSAwLjUpKSsKICAgIGdlb21fcG9pbnQoZGF0YT1yZXN1bHRzWzE6MTAsXSxhZXMoeCA9IGxvZzJGb2xkQ2hhbmdlLCB5ID0gLWxvZzEwKHBhZGopLCBjb2xvcj0icmVkIiwgc2l6ZT0xLjUsIGFscGhhPTAuNCkpKwogICAgc2NhbGVfY29sb3JfbWFudWFsKHZhbHVlcz1jKCdncmF5JywncmVkJykpCgpnZ3NhdmUoCiAgICAidm9sY2Fub3Bsb3QucG5nIiwKICAgIHBsb3QgPSBsYXN0X3Bsb3QoKSwKICAgIGRwaT02MDApCgojbm9ybWFsaXplIHRoZSByYXdjb3VudHMsIGFuZCB3cml0ZSBhIHRhYmxlIHdpdGggdGhlIG5vcm1hbGl6ZWQgY291bnRzCmRub3JtYWxhbmR0dW1vcl9WU0QgPC12YXJpYW5jZVN0YWJpbGl6aW5nVHJhbnNmb3JtYXRpb24oZG5vcm1hbGFuZHR1bW9yKQp3cml0ZS5jc3YoYXNzYXkoZG5vcm1hbGFuZHR1bW9yX1ZTRCksIGZpbGU9Im5vcm1hbGl6ZWRfY291bnRzLmNzdiIsIHJvdy5uYW1lcyA9IHJhd2NvdW50cyRYKQoKI21ha2UgYSBzbWFsbCBQQ0EgcGxvdCBmb3IgYW4gaW5pdGlhbCBvdmVydmlldwpkbm9ybWFsYW5kdHVtb3JfVlNECnBsb3RQQ0EoZG5vcm1hbGFuZHR1bW9yX1ZTRCxudG9wPTMwMDAwLGludGdyb3VwPSJ0cmVhdG1lbnQiKSsgdGhlbWVfYncoKQoKZ2dzYXZlKAogICAgIlBDQXBsb3QucG5nIiwKICAgIHBsb3QgPSBsYXN0X3Bsb3QoKSwKICAgIGRwaT02MDApCgpwcmludCgiZG9uZSIpCgpgYGA=
