# Supplementary material for: Single-Cell Transcriptomics Reveals the Expression of Aging- and Senescence-Associated Genes in Distinct Cancer Cell Populations
Source: Cells. 2021 Nov 11;10(11):3126. doi: 10.3390/cells10113126 (PMC8623328; doi:10.3390/cells10113126)
Supplement: Supplementary file 1 [file cells-10-03126-s001.zip › single_cell_RNA_Seq_MM.nb.html]

R Notebook single\_cell\_RNA\_Seq


Code 

- Show All Code
- Hide All Code
- Download Rmd

# R Notebook single\_cell\_RNA\_Seq


```
##install BiocManager, if not existent
packages = c("BiocManager") 
package.check <- lapply(
    packages,
    FUN = function(x) {
        if (!require(x, character.only = TRUE)) {
            install.packages(x, dependencies = TRUE)
            library(x, character.only = TRUE)
        }
    }
)

packages = c("Seurat", "swne", "monocle", "DDRTree", "Nebulosa")

#if the needed packages do not exist, they will be installed:
package.check <- lapply(
    packages,
    FUN = function(x) {
        if (!require(x, character.only = TRUE)) {
            BiocManager::install(x, dependencies = TRUE)
            library(x, character.only = TRUE)
        }
    }
)

#create a new folder for this dataset, and set the working directory to this folder
if (file.exists("GSE175385")) {
    ""
} else {
    dir.create("GSE175385")
}

setwd("GSE175385")

if (file.exists("GSE175385_Seurat_object.Rdata")) {
    ""
} else {
#get the raw_counts_file from GEO, download them and untar them
options(timeout=100000000000)

file.exists("leaflet.R")
url="https://www.ncbi.nlm.nih.gov/geo/download/?acc=GSE175385&format=file&file=GSE175385%5FSeurat%5Fobject%2ERdata%2Egz"
download.file(url, "GSE175385_Seurat_object.Rdata.gz")
gunzip("GSE175385_Seurat_object.Rdata.gz", remove=FALSE)
}

#load the Seurat file
load("GSE175385_Seurat_object.Rdata")

#prepare the Seurat file, keep just the MM samples
super.merge_NKandBremove@meta.data$origin2 <- ""

super.merge_NKandBremove@meta.data$origin2 <- substr(super.merge_NKandBremove$origin, 1, 2)

MM_seurat <- subset(super.merge_NKandBremove, subset=origin2=="MM")

#swne-Plot, Panel A
#add the previously found CML key genes
cml_genes <- c("ABI3","AKR1B1","AKT3","APOL3","ASF1A","BAG3","BLK","CDC25B","CDKN1B","CYP1B1","ETS1","GPR155","IL7R","KCNA3","MAP4K1","MPEG1","NOG","OPTN","PARP1","PRKCH","SMAD3","SUN1","TXNIP","VENTX")

#create a swne embedding
swne.embedding <- RunSWNE(MM_seurat, k = 16, genes.embed = cml_genes)

#create the correct color code
clusters <- MM_seurat$origin2
cluster.colors <- ExtractSWNEColors(swne.embedding, clusters, seed = 42)

cluster.colors[["MM"]] <- "#C00000"

#create the first figure with the correct color code
pdf("panel_A.pdf")
PlotSWNE(swne.embedding, alpha.plot = 0.4, sample.groups = clusters, do.label = F, label.size = 0.1, pt.size = 1.5, show.legend = F, seed = NULL) + scale_color_manual(values = cluster.colors)
dev.off()

#Monocle: Pseudotime trajectory and heatmap, Panel B and C
data <- as(as.matrix(MM_seurat@assays$RNA@data), 'sparseMatrix')
pd <- new('AnnotatedDataFrame', data = MM_seurat@meta.data)
fData <- data.frame(gene_short_name = row.names(data), row.names = row.names(data))
fd <- new('AnnotatedDataFrame', data = fData)
HSMM <- newCellDataSet(data, phenoData = pd, featureData = fd, lowerDetectionLimit = 0.5, expressionFamily = uninormal())

MM_seurat <- NormalizeData(MM_seurat)
MM_seurat@active.assay <- "RNA"
MM_seurat <- FindVariableFeatures(MM_seurat, selection.method = "vst", nfeatures = 2000)
var_genes <- MM_seurat[["RNA"]]@var.features

HSMM <- setOrderingFilter(HSMM, var_genes)

HSMM <- reduceDimension(HSMM,norm_method="none", reduction_method="DDRTree", max_components=4, scaling=TRUE,verbose=TRUE, pseudo_expr=0)

HSMM <- orderCells(HSMM)

pdf("panel_B.pdf") 
plot_cell_trajectory(HSMM, color_by = "Pseudotime", theta = 210, show_branch_points = FALSE, show_tree = TRUE, cell_size = 0.01) + scale_color_gradient(low="#C00000", high = "black")
dev.off()

marker_genes <- row.names(subset(fData(HSMM), gene_short_name %in% cml_genes))
diff_test_res <- differentialGeneTest(HSMM[marker_genes,], fullModelFormulaStr = "~sm.ns(Pseudotime)")
sig_gene_names <- row.names(subset(diff_test_res, qval < 0.1))

pdf("panel_C.pdf")
plot_pseudotime_heatmap(HSMM[sig_gene_names,],
                        num_clusters = 3,
                        cores = 2,
                        show_rownames = T)
dev.off()

MM_seurat <- RunTSNE(MM_seurat, dims = 1:40, reduction = "pca", check_duplicates=FALSE)
mm_selection <- c("CDKN1B", "CDC25B", "KCNA3", "AKT3", "OPTN", "BAG3", "SUN1", "AKR1B1")

pdf("panel_D.pdf")
plot_density(MM_seurat, mm_selection, reduction= "tsne", joint="TRUE") * scale_color_gradient(low="grey", high = "#C00000") * theme(legend.position = "")
dev.off()

print("done")
```


LS0tCnRpdGxlOiAiUiBOb3RlYm9vayBzaW5nbGVfY2VsbF9STkFfU2VxIgpvdXRwdXQ6IGh0bWxfbm90ZWJvb2sKLS0tCmBgYHtyfQojI2luc3RhbGwgQmlvY01hbmFnZXIsIGlmIG5vdCBleGlzdGVudApwYWNrYWdlcyA9IGMoIkJpb2NNYW5hZ2VyIikgCnBhY2thZ2UuY2hlY2sgPC0gbGFwcGx5KAogICAgcGFja2FnZXMsCiAgICBGVU4gPSBmdW5jdGlvbih4KSB7CiAgICAgICAgaWYgKCFyZXF1aXJlKHgsIGNoYXJhY3Rlci5vbmx5ID0gVFJVRSkpIHsKICAgICAgICAgICAgaW5zdGFsbC5wYWNrYWdlcyh4LCBkZXBlbmRlbmNpZXMgPSBUUlVFKQogICAgICAgICAgICBsaWJyYXJ5KHgsIGNoYXJhY3Rlci5vbmx5ID0gVFJVRSkKICAgICAgICB9CiAgICB9CikKCnBhY2thZ2VzID0gYygiU2V1cmF0IiwgInN3bmUiLCAibW9ub2NsZSIsICJERFJUcmVlIiwgIk5lYnVsb3NhIikKCiNpZiB0aGUgbmVlZGVkIHBhY2thZ2VzIGRvIG5vdCBleGlzdCwgdGhleSB3aWxsIGJlIGluc3RhbGxlZDoKcGFja2FnZS5jaGVjayA8LSBsYXBwbHkoCiAgICBwYWNrYWdlcywKICAgIEZVTiA9IGZ1bmN0aW9uKHgpIHsKICAgICAgICBpZiAoIXJlcXVpcmUoeCwgY2hhcmFjdGVyLm9ubHkgPSBUUlVFKSkgewogICAgICAgICAgICBCaW9jTWFuYWdlcjo6aW5zdGFsbCh4LCBkZXBlbmRlbmNpZXMgPSBUUlVFKQogICAgICAgICAgICBsaWJyYXJ5KHgsIGNoYXJhY3Rlci5vbmx5ID0gVFJVRSkKICAgICAgICB9CiAgICB9CikKCiNjcmVhdGUgYSBuZXcgZm9sZGVyIGZvciB0aGlzIGRhdGFzZXQsIGFuZCBzZXQgdGhlIHdvcmtpbmcgZGlyZWN0b3J5IHRvIHRoaXMgZm9sZGVyCmlmIChmaWxlLmV4aXN0cygiR1NFMTc1Mzg1IikpIHsKICAgICIiCn0gZWxzZSB7CiAgICBkaXIuY3JlYXRlKCJHU0UxNzUzODUiKQp9CgpzZXR3ZCgiR1NFMTc1Mzg1IikKCmlmIChmaWxlLmV4aXN0cygiR1NFMTc1Mzg1X1NldXJhdF9vYmplY3QuUmRhdGEiKSkgewogICAgIiIKfSBlbHNlIHsKI2dldCB0aGUgcmF3X2NvdW50c19maWxlIGZyb20gR0VPLCBkb3dubG9hZCB0aGVtIGFuZCB1bnRhciB0aGVtCm9wdGlvbnModGltZW91dD0xMDAwMDAwMDAwMDApCgpmaWxlLmV4aXN0cygibGVhZmxldC5SIikKdXJsPSJodHRwczovL3d3dy5uY2JpLm5sbS5uaWguZ292L2dlby9kb3dubG9hZC8/YWNjPUdTRTE3NTM4NSZmb3JtYXQ9ZmlsZSZmaWxlPUdTRTE3NTM4NSU1RlNldXJhdCU1Rm9iamVjdCUyRVJkYXRhJTJFZ3oiCmRvd25sb2FkLmZpbGUodXJsLCAiR1NFMTc1Mzg1X1NldXJhdF9vYmplY3QuUmRhdGEuZ3oiKQpndW56aXAoIkdTRTE3NTM4NV9TZXVyYXRfb2JqZWN0LlJkYXRhLmd6IiwgcmVtb3ZlPUZBTFNFKQp9CgojbG9hZCB0aGUgU2V1cmF0IGZpbGUKbG9hZCgiR1NFMTc1Mzg1X1NldXJhdF9vYmplY3QuUmRhdGEiKQoKI3ByZXBhcmUgdGhlIFNldXJhdCBmaWxlLCBrZWVwIGp1c3QgdGhlIE1NIHNhbXBsZXMKc3VwZXIubWVyZ2VfTkthbmRCcmVtb3ZlQG1ldGEuZGF0YSRvcmlnaW4yIDwtICIiCgpzdXBlci5tZXJnZV9OS2FuZEJyZW1vdmVAbWV0YS5kYXRhJG9yaWdpbjIgPC0gc3Vic3RyKHN1cGVyLm1lcmdlX05LYW5kQnJlbW92ZSRvcmlnaW4sIDEsIDIpCgpNTV9zZXVyYXQgPC0gc3Vic2V0KHN1cGVyLm1lcmdlX05LYW5kQnJlbW92ZSwgc3Vic2V0PW9yaWdpbjI9PSJNTSIpCgojc3duZS1QbG90LCBQYW5lbCBBCiNhZGQgdGhlIHByZXZpb3VzbHkgZm91bmQgQ01MIGtleSBnZW5lcwpjbWxfZ2VuZXMgPC0gYygiQUJJMyIsIkFLUjFCMSIsIkFLVDMiLCJBUE9MMyIsIkFTRjFBIiwiQkFHMyIsIkJMSyIsIkNEQzI1QiIsIkNES04xQiIsIkNZUDFCMSIsIkVUUzEiLCJHUFIxNTUiLCJJTDdSIiwiS0NOQTMiLCJNQVA0SzEiLCJNUEVHMSIsIk5PRyIsIk9QVE4iLCJQQVJQMSIsIlBSS0NIIiwiU01BRDMiLCJTVU4xIiwiVFhOSVAiLCJWRU5UWCIpCgojY3JlYXRlIGEgc3duZSBlbWJlZGRpbmcKc3duZS5lbWJlZGRpbmcgPC0gUnVuU1dORShNTV9zZXVyYXQsIGsgPSAxNiwgZ2VuZXMuZW1iZWQgPSBjbWxfZ2VuZXMpCgojY3JlYXRlIHRoZSBjb3JyZWN0IGNvbG9yIGNvZGUKY2x1c3RlcnMgPC0gTU1fc2V1cmF0JG9yaWdpbjIKY2x1c3Rlci5jb2xvcnMgPC0gRXh0cmFjdFNXTkVDb2xvcnMoc3duZS5lbWJlZGRpbmcsIGNsdXN0ZXJzLCBzZWVkID0gNDIpCgpjbHVzdGVyLmNvbG9yc1tbIk1NIl1dIDwtICIjQzAwMDAwIgoKI2NyZWF0ZSB0aGUgZmlyc3QgZmlndXJlIHdpdGggdGhlIGNvcnJlY3QgY29sb3IgY29kZQpwZGYoInBhbmVsX0EucGRmIikKUGxvdFNXTkUoc3duZS5lbWJlZGRpbmcsIGFscGhhLnBsb3QgPSAwLjQsIHNhbXBsZS5ncm91cHMgPSBjbHVzdGVycywgZG8ubGFiZWwgPSBGLCBsYWJlbC5zaXplID0gMC4xLCBwdC5zaXplID0gMS41LCBzaG93LmxlZ2VuZCA9IEYsIHNlZWQgPSBOVUxMKSArIHNjYWxlX2NvbG9yX21hbnVhbCh2YWx1ZXMgPSBjbHVzdGVyLmNvbG9ycykKZGV2Lm9mZigpCgojTW9ub2NsZTogUHNldWRvdGltZSB0cmFqZWN0b3J5IGFuZCBoZWF0bWFwLCBQYW5lbCBCIGFuZCBDCmRhdGEgPC0gYXMoYXMubWF0cml4KE1NX3NldXJhdEBhc3NheXMkUk5BQGRhdGEpLCAnc3BhcnNlTWF0cml4JykKcGQgPC0gbmV3KCdBbm5vdGF0ZWREYXRhRnJhbWUnLCBkYXRhID0gTU1fc2V1cmF0QG1ldGEuZGF0YSkKZkRhdGEgPC0gZGF0YS5mcmFtZShnZW5lX3Nob3J0X25hbWUgPSByb3cubmFtZXMoZGF0YSksIHJvdy5uYW1lcyA9IHJvdy5uYW1lcyhkYXRhKSkKZmQgPC0gbmV3KCdBbm5vdGF0ZWREYXRhRnJhbWUnLCBkYXRhID0gZkRhdGEpCkhTTU0gPC0gbmV3Q2VsbERhdGFTZXQoZGF0YSwgcGhlbm9EYXRhID0gcGQsIGZlYXR1cmVEYXRhID0gZmQsIGxvd2VyRGV0ZWN0aW9uTGltaXQgPSAwLjUsIGV4cHJlc3Npb25GYW1pbHkgPSB1bmlub3JtYWwoKSkKCk1NX3NldXJhdCA8LSBOb3JtYWxpemVEYXRhKE1NX3NldXJhdCkKTU1fc2V1cmF0QGFjdGl2ZS5hc3NheSA8LSAiUk5BIgpNTV9zZXVyYXQgPC0gRmluZFZhcmlhYmxlRmVhdHVyZXMoTU1fc2V1cmF0LCBzZWxlY3Rpb24ubWV0aG9kID0gInZzdCIsIG5mZWF0dXJlcyA9IDIwMDApCnZhcl9nZW5lcyA8LSBNTV9zZXVyYXRbWyJSTkEiXV1AdmFyLmZlYXR1cmVzCgpIU01NIDwtIHNldE9yZGVyaW5nRmlsdGVyKEhTTU0sIHZhcl9nZW5lcykKCkhTTU0gPC0gcmVkdWNlRGltZW5zaW9uKEhTTU0sbm9ybV9tZXRob2Q9Im5vbmUiLCByZWR1Y3Rpb25fbWV0aG9kPSJERFJUcmVlIiwgbWF4X2NvbXBvbmVudHM9NCwgc2NhbGluZz1UUlVFLHZlcmJvc2U9VFJVRSwgcHNldWRvX2V4cHI9MCkKCkhTTU0gPC0gb3JkZXJDZWxscyhIU01NKQoKcGRmKCJwYW5lbF9CLnBkZiIpIApwbG90X2NlbGxfdHJhamVjdG9yeShIU01NLCBjb2xvcl9ieSA9ICJQc2V1ZG90aW1lIiwgdGhldGEgPSAyMTAsIHNob3dfYnJhbmNoX3BvaW50cyA9IEZBTFNFLCBzaG93X3RyZWUgPSBUUlVFLCBjZWxsX3NpemUgPSAwLjAxKSArIHNjYWxlX2NvbG9yX2dyYWRpZW50KGxvdz0iI0MwMDAwMCIsIGhpZ2ggPSAiYmxhY2siKQpkZXYub2ZmKCkKCm1hcmtlcl9nZW5lcyA8LSByb3cubmFtZXMoc3Vic2V0KGZEYXRhKEhTTU0pLCBnZW5lX3Nob3J0X25hbWUgJWluJSBjbWxfZ2VuZXMpKQpkaWZmX3Rlc3RfcmVzIDwtIGRpZmZlcmVudGlhbEdlbmVUZXN0KEhTTU1bbWFya2VyX2dlbmVzLF0sIGZ1bGxNb2RlbEZvcm11bGFTdHIgPSAifnNtLm5zKFBzZXVkb3RpbWUpIikKc2lnX2dlbmVfbmFtZXMgPC0gcm93Lm5hbWVzKHN1YnNldChkaWZmX3Rlc3RfcmVzLCBxdmFsIDwgMC4xKSkKCnBkZigicGFuZWxfQy5wZGYiKQpwbG90X3BzZXVkb3RpbWVfaGVhdG1hcChIU01NW3NpZ19nZW5lX25hbWVzLF0sCiAgICAgICAgICAgICAgICAgICAgICAgIG51bV9jbHVzdGVycyA9IDMsCiAgICAgICAgICAgICAgICAgICAgICAgIGNvcmVzID0gMiwKICAgICAgICAgICAgICAgICAgICAgICAgc2hvd19yb3duYW1lcyA9IFQpCmRldi5vZmYoKQoKTU1fc2V1cmF0IDwtIFJ1blRTTkUoTU1fc2V1cmF0LCBkaW1zID0gMTo0MCwgcmVkdWN0aW9uID0gInBjYSIsIGNoZWNrX2R1cGxpY2F0ZXM9RkFMU0UpCm1tX3NlbGVjdGlvbiA8LSBjKCJDREtOMUIiLCAiQ0RDMjVCIiwgIktDTkEzIiwgIkFLVDMiLCAiT1BUTiIsICJCQUczIiwgIlNVTjEiLCAiQUtSMUIxIikKCnBkZigicGFuZWxfRC5wZGYiKQpwbG90X2RlbnNpdHkoTU1fc2V1cmF0LCBtbV9zZWxlY3Rpb24sIHJlZHVjdGlvbj0gInRzbmUiLCBqb2ludD0iVFJVRSIpICogc2NhbGVfY29sb3JfZ3JhZGllbnQobG93PSJncmV5IiwgaGlnaCA9ICIjQzAwMDAwIikgKiB0aGVtZShsZWdlbmQucG9zaXRpb24gPSAiIikKZGV2Lm9mZigpCgpwcmludCgiZG9uZSIpCgpgYGAK
